# A cognitive chameleon: Lessons from a novel *MAPT* mutation case

**DOI:** 10.1080/13554794.2013.826697

**Published:** 2013-09-02

**Authors:** Yuying Liang, Elizabeth Gordon, Jonathan Rohrer, Laura Downey, Rohan de Silva, Hans Rolf Jäger, Jennifer Nicholas, Marc Modat, M. Jorge Cardoso, Colin Mahoney, Jason Warren, Martin Rossor, Nick Fox, Diana Caine

**Affiliations:** ^a^ Dementia Research Centre, Department of Neurodegenerative Diseases, UCL Institute of Neurology, LondonWC1N 3BG, UK; ^b^ Reta Lila Weston Institute, Department of Molecular Neuroscience, UCL Institute of Neurology, LondonWC1N 1PJ, UK; ^c^ Lysholm Department of Neuroradiology, National Hospital for Neurology and Neurosurgery, LondonWC1N 3BG, UK; ^d^ Department of Medical Statistics, London School of Hygiene and Tropical Medicine, Keppel Street, LondonWC1E 7HT, UK; ^e^ Centre for Medical Image Computing, UCL, Gower Street, LondonWC1E 6BT, UK; ^f^ Department of Neuropsychology, National Hospital for Neurology and Neurosurgery, LondonWC1N 3BG, UK

**Keywords:** Episodic memory, Semantic memory, Familial Alzheimer’s disease, *MAPT* mutations, Frontotemporal dementia, Autobiographical memory, Brain networks

## Abstract

We report a case of frontotemporal dementia caused by a novel *MAPT* mutation (Q351R) with a remarkably long amnestic presentation mimicking familial Alzheimer’s disease. Longitudinal clinical, neuropsychological and imaging data provide convergent evidence for predominantly bilateral anterior medial temporal lobe involvement consistent with previously established neuroanatomical signatures of *MAPT* mutations. This case supports the notion that the neural network affected in *MAPT* mutations is determined to a large extent by the underlying molecular pathology. We discuss the diagnostic significance of anomia in the context of atypical amnesia and the impact of impaired episodic and semantic memory systems on autobiographical memory.

Frontotemporal dementia (FTD) is a common cause of young onset dementia (Harvey, Skelton-Robinson, & Rossor, 2003; Ratnavalli, Brayne, Dawson, & Hodges, 2002). Around a third to a half of patients with FTD have a family history with approximately 10–40% following an autosomal dominant inheritance pattern (Bird et al., 2003; Chow, Miller, Hayashi, & Geschwind, 1999; Rohrer et al., 2009). *MAPT, PGN*, and C*9ORF72* mutations have been identified as three major genetic causes. Studying patients with genetic forms of FTD can reveal potential relationships between specific molecular pathologies and clinical phenotypes which could in turn be helpful in elucidating the underlying disease mechanisms. *MAPT* mutations typically present with behavioral changes with subsequent emergence of semantic deficits, memory decline, and motor features (Rohrer & Warren, 2011). Occasionally one of the nonbehavioral features predominates, resulting in patients receiving an alternative diagnosis initially. We report a patient who presented with a remarkably long history of amnesia with early features of semantic loss followed later by behavioral changes.

**TABLE 1:** Select neuropsychology results at baseline, 8-year and 13-year follow-up visits

Session	Full-scale IQ	Recognition memory test (words)	Recognition memory test (faces)	WMS-delayed story recall	Rey complex figure recall	
Baseline	108	36 (<5%)	35 (<5%)	−	−	
8 yr	102	32 (<5%)	20 (<5%)	0 (<1%)	0 (<1%)	
13 yr	86	27 (<5%)	25 (<5%)	0 (<1%)	0 (<1%)	
*Session*	*Graded naming test*	*Famousfaces*	*Categoryfluency*	*Letterfluency*	*Synonym-matching*	
					*Concrete*	*Abstract*
Baseline	5 (5%)	−	17 (25%)	21 (>90%)	−	−
8 yr	2 (<1%)	2	9 (<10%)	17 (50–70%)	−	−
13 yr	0 (<1%)	0	4 (<1%)	15 (44%)	16 (<1%)	18 (10%)
*Session*	*Trail making test B*	*Stroop inhibition*	*Cognitive estimate*			
Baseline	−	−	3 (50%)			
8 yr	140′′ (10–25%)	20–24%	−			
13 yr	146′′ (10–25%)	25–50%	15 (<1%)			

## CASE DESCRIPTION

CW (initials changed to preserve anonymity) is a right-handed lady who was born at term and reached normal developmental milestones. She completed a university degree and worked at a relatively senior level in the public sector. In her mid-40s, her work performance deteriorated. Around the same time, she became irritable and argumentative leading to strained relationships with her partner and children. With hindsight, her now former partner reports a subtle decline in her memory from her late thirties.

She was first seen in our cognitive disorders clinic at the age of 49 years old, with a principal complaint of poor memory. She was forgetting appointments and had started to keep a diary. She had been subjected to three separate capability assessments at work. In addition to relationship difficulties, there were also financial problems. She had had some depressive symptoms including low mood, an exaggeration of premorbid pessimism, and diminished interest and drive. General and neurological examinations were normal.

**Figure 1.**
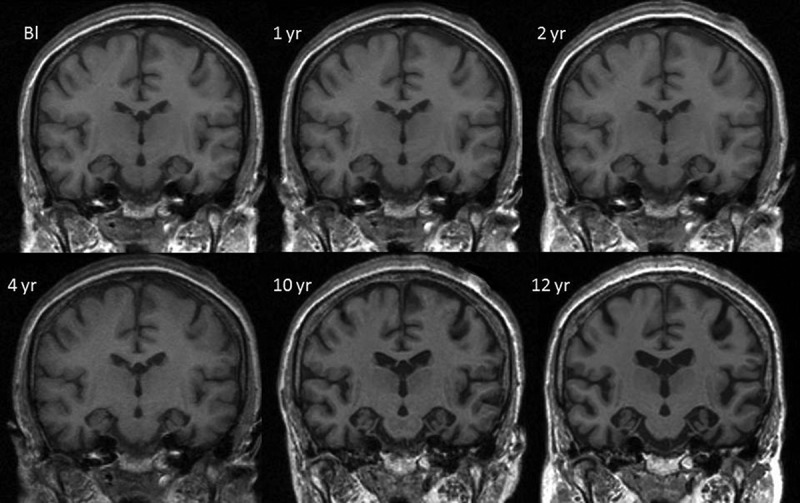
Mid-temporal axial volumetric T1-weighted MR images acquired at initial presentation and subsequent repeat visits (1-, 2-, 4-, 10-, and 12-year follow-up). All repeat images have undergone 12 degrees of freedom registration to spatially align them to the baseline (Bl).

As detailed in Table 1, the first neuropsychological assessment showed a mild reduction in general intellectual function (FSIQ108 compared with NART-estimated FSIQ 118) (Nelson, 1982; Wechsler, 1981) with impaired performance on both verbal and visual versions of the recognition memory test (RMT both <5th percentile) (Warrington, 1984). Confrontation naming was poor (graded naming test (GNT): 5th percentile; Oldfield naming test: 25–50th percentile) (McKenna & Warrington, 1983; Oldfield & Wingfield, 1965) which, together with slightly reduced category fluency (animal names: 25th percentile) (Spreen & Strauss, 1998), hinted at early semantic impairment, especially in comparison with preserved phonemic fluency (“s”: >90th percentile) (Spreen & Strauss, 1998). The latter indicated preserved executive function as did sound cognitive estimates (50th percentile) (Shallice & Evans, 1978). Visual processing was intact (VOSP incomplete letters: >5th percentile) (Warrington & James, 1991). Notwithstanding some reduced scores, inconsistencies were noted between poor naming, fluent spontaneous speech, and preserved vocabulary score on the WAIS-R; and between poor memory scores and her ability to provide details regarding her present circumstances. In addition, she showed no implicit learning on the Gollin figures test (Gollin, 1960). These factors, in the context of her relatively young age and reported changes in mood, suggested depression and additional functional component as possible causes for her cognitive problems. However, her MRI brain scan (Figure 1) showed small medial temporal lobes for age.

## DISEASE PROGRESSION

The clinical impression that depression was a significant contributor to her cognitive complaints was revised 2 years later when, at the age of 51 years old, CW’s memory had deteriorated significantly and she had had to give up work. She would now frequently forget the previous day’s events and had difficulty finding familiar places while driving. Treatment with Citalopram for low mood and anxiety had had no impact on her cognitive function. At this point, a more detailed family history emerged. CW’s father had developed apathy at the age of 39 years old followed by progressive memory decline. He died at the age of 55 years old. Two of his three siblings were similarly affected.

Further investigations were performed. Cerebrospinal fluid was normal for cell counts, protein and glucose. S100 was 0.96 ng/ml (reference range: 99% below 0.55 ng/ml). Oligoclonal bands were negative in the CSF and serum. Tau and Aβ_42_ levels were not available. EEG demonstrated normal alpha rhythm with no epileptic activity. Repeat MRI brain appeared to show stable intracranial appearances but was now reported to be consistent with Alzheimer’s disease (AD) (Figure 1). A provisional diagnosis of AD was made. On account of the strong family history, she was screened for mutations in the *PSEN1*, 2 and *APP* genes which were all negative.

Continuing cognitive deterioration followed in the succeeding years such that by 57 years of age, 8 years after the initial presentation, she required a support worker to assist with daily tasks. In addition to poor memory, occasional word-finding difficulties, the onset of a sweet tooth, and some weight gain were reported. She tended to oversleep, even during the day. She was no longer oriented in time. There was further decline in naming (GNT: 2/30, <1st percentile; Oldfield Naming test: 16/30, 10–25th percentile) and semantic fluency (animal names: <10th percentile) with a milder deterioration in phonemic fluency (50–70th percentile). There was mild executive impairment (trail making part B: 10–25th percentile; stroop inhibition: 20–24th percentile) (Table 1). At this time, the profile comprising profound episodic and semantic memory loss together with milder executive problems was still thought to be consistent with a diagnosis of early onset AD. There was now the intimation of behavioral changes including some disinhibition on the letter fluency task in the form of sex-related words in addition to the change in eating habits.

Clear behavioral symptoms emerged when CW was 61 years old. She had become disinhibited and frequently gave strangers her phone number and address. She was increasingly apathetic and would lie in bed all day unless prompted and needed encouragement even to attend social functions that she continued to enjoy. She had become less empathic. Her intake of sweet food had increased and she had developed musicophilia. The latter took the form of listening to certain types of music continually. She was started on Donepezil to no great effect.

At this point, her baseline MRI and 4- and 10-year repeat scans were spatially aligned using a 12 degrees of freedom (d.o.f.) registration as part of her radiological review. This procedure allows regions of cortical change between timepoints to be quantified using the boundary shift integral (BSI) and aids in visual assessment of volume loss (Freeborough & Fox, 1997; Leung et al., 2010). Review of these registered images indicated a progression in the anterior–posterior gradient of volume loss across the medial temporal lobes with disproportionate loss anteriorly. This raised the possible diagnosis of familial FTD due to a tau mutation. She underwent another lumbar puncture and the CSF analysis revealed a tau level of 429 pg/ml (suggestive of neurodegeneration) (local reference range: abnormal >400 pg/ml) and an Aβ_42_ level of 723 pg/ml (non-AD like) (local reference range: AD profile if <300 pg/ml). Screening for *MAPT* mutations revealed a novel mutation in exon 12 with a single heterozygous nucleotide change c.1052A >G.

Presently, 13 years after initial presentation, CW is profoundly amnesic. She is very repetitive and her conversation is impoverished in content. She gives the same highly stereotyped and very restricted account of her childhood to everyone she meets.

The latest neuropsychology assessment at the age of 62 years showed general cognitive decline (WASI-II FSIQ 86) (Wechsler, 2011). Working memory as measured by digit span remained within normal limits scaled score 10) (Wechsler, 1987). Recognition memory was at chance. She had no recall of a short story in immediate or delayed recall conditions. Naming scores were progressively lower across all tests (GNT now 0/30, Boston Naming Test 18/30) (Kaplan, Goodglass, & Weintraub, 1983), with worsening semantic deterioration (animal names: 4, vegetable names: 0) also a feature. Phonemic fluency was further reduced but executive function *per se* remained otherwise relatively stable. On formal examination, recall of both personal semantic and autobiographic incidents was impaired (Kopelman, Wilson, & Baddeley, 1989), the latter more dramatically so (Table 2). Notably, posterior function remained relatively intact (VOSP object decision: >5% cut-off; complex figure copy: 25–50%). The profile thus remained one of profound episodic and semantic memory loss with less marked executive dysfunction but now in the context of striking behavioral change.

**TABLE 2:** Autobiographical memory interview at the age of 61 years old (12-year follow-up visit)

Total score summary	Personal semantic	Autobiographical incidents
Section A childhood	5.5/21	0/9
Section B early adult life	10/21	2/9
Section C recent Life	4.5/21	0/9
	20/63 (32%)	2/27 (7%)
Cut-off	<47	<12

Neurologically she now has evidence of mild asymmetric parkinsonism and pyramidal signs. There is a mild resting tremor in her left hand associated with bradykinesia. She has pathologically brisk reflexes in her right arm and leg, Hoffman’s sign in her right hand and an extensor right planter. Orofacial and limb praxis are normal.

## NEUROIMAGING

**Figure 2.**
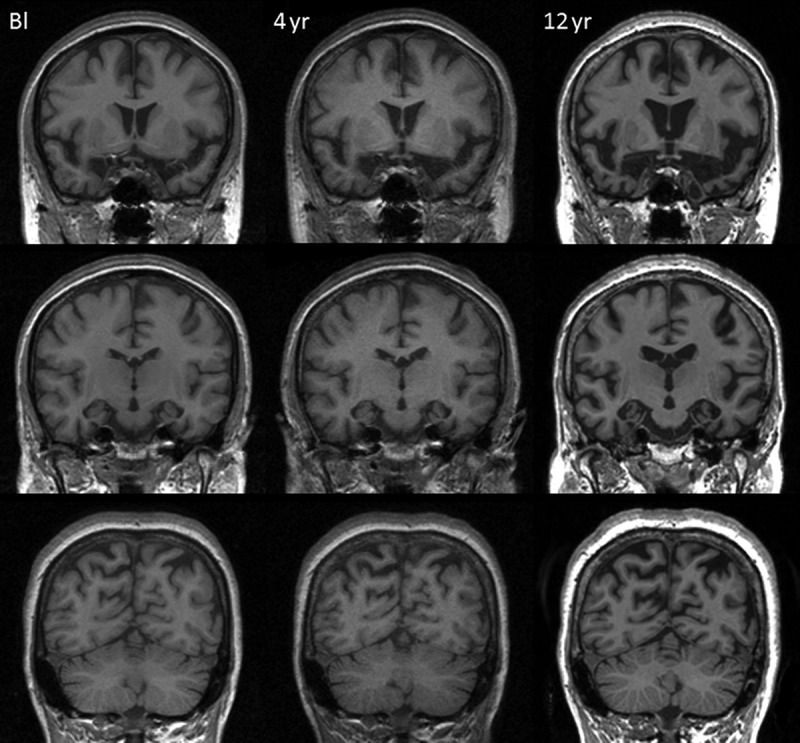
Spatially aligned coronal MR images from baseline (Bl), 4-year and 12-year follow-up visits demonstrating progressive atrophy affecting frontal, temporal, and parietal lobes.

As noted earlier, the first MRI brain scan at presentation (aged 49) was thought to show bilateral medial temporal atrophy. More recent blinded radiological review of these images also reported frontal and parietal lobe atrophy (Figure 2). Subsequent longitudinal MRI scans demonstrated progressive global cerebral atrophy with particularly striking changes in the anterior, inferior, and medial aspects of the temporal lobes (Figures 1 and 2).

**Figure 3.**
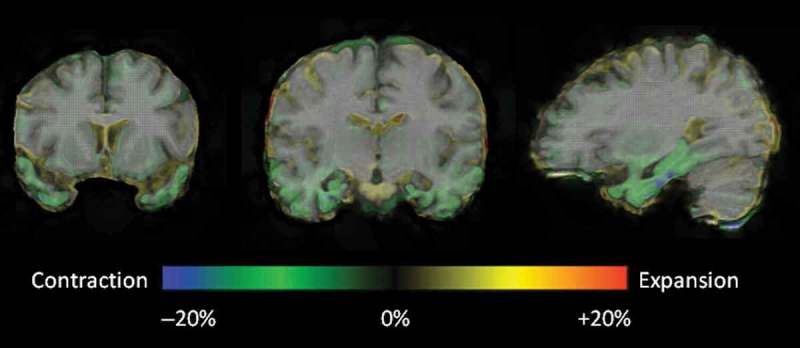
Representative coronal and sagittal MRI slices with voxel deformation mapping overlay, over an interval of 4 years post-presentation. These demonstrate relatively focal bilateral contraction (green/blue = volume loss) in the temporal lobes, particularly involving the temporal poles, parahippocampal and fusiform gyri, with an anterior–posterior gradient. [To view this figure in colour, please see the online version of this Journal.]

The pattern of these early temporal lobe changes are more clearly demonstrated by whole brain fluid registration (Figure 3). This method involves nonlinear warping of each scan to match the baseline scan, generating a deformation field that allows visualization of voxel-level expansion and contraction (Freeborough & Fox, 1998). These images show clear evidence of progressive atrophy in the temporal lobes, frontal gyri, and the insular cortices early in the disease process, before it was detectable during routine radiological assessment.

Given the novel mutation and the atypical clinical presentation, we compared CW’s scans with those of 9*MAPT* cases in our FTD cohort and 13 healthy controls matched for age and gender. All images underwent non-uniformity correction, manual whole-brain delineation, and affine 12 d.o.f. registration to quantify longitudinal whole brain change. Hippocampal volumes were derived using a template based method for automated segmentations (Cardoso et al., 2013) and used to investigate longitudinal hippocampal volume change for CW and the nine *MAPT* mutation cases with longitudinal imaging. We generated a head size measure by estimating total intracranial volumes (TIV) from the summation of the volumes of gray matter, white matter, and cerebral spinal fluid using the segmentation toolbox in Statistical Parametric Mapping version 8 (Wellcome Trust Centre for Neuroimaging, London, UK, http://www.fil.ion.ucl.ac.uk/spm; Leung et al., 2010).

**Figure 4.**
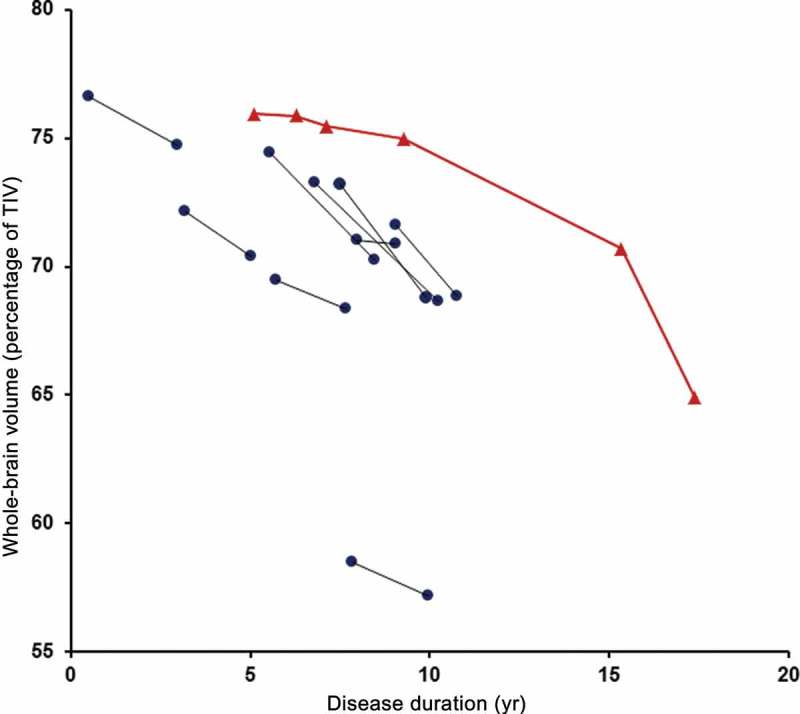
Longitudinal whole brain volumes (as percentage of total intracranial volume) for • *MAPT* mutation cases and ▴ CW against disease duration. [To view this figure in colour, please see the online version of this Journal.]

**Figure 5.**
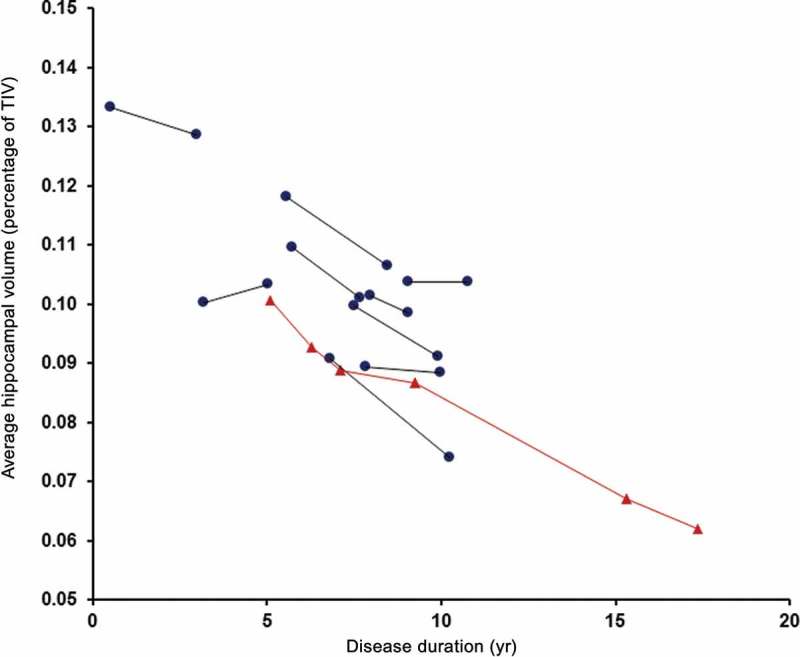
Longitudinal average hippocampal volumes (as percentage of total intracranial volume) for • *MAPT* mutation cases and ▴ CW against disease duration. [To view this figure in colour, please see the online version of this Journal.]

CW’s early global longitudinal profile appeared very stable with volumetric analysis revealing whole-brain volumes that ranged from 1017 mm^3^ (75.9% of TIV) at baseline to 1004 mm^3^ (75.0% of TIV) at 4-year follow-up (Figure 4). Between these two time points, her average annual loss of 0.28% of baseline whole-brain volume was comparable to our controls (mean = 0.27%, SD = 0.32) T (12) = 0.05, *p* = .96 (Crawford & Howell, 1998) and was also consistent with previously published rates for healthy controls (mean 0.32%, 95% CI 0.1–0.54%) (Scahill et al., 2003). Compared to the other *MAPT* mutation cases, her whole-brain atrophy rate at this early stage was toward the lower end of the range but was not significantly different (mean = 1.44%, SD = 0.76, range = 0.20–2.53%) T (8) = −1.45, *p* = .19. When compared to three of the *MAPT* cases of similar disease duration, CW’s whole-brain atrophy rate was again in the lower range. Her annualized hippocampal rate of change over the same time period (3.35%) was significantly higher than our controls (mean = 0.30%, SD = 1.30) T (12) = 2.26, *p* = .04 and was toward the higher end of the range compared to the other *MAPT* mutation cases but again not significantly different (mean = 2.16%, SD = 2.25, range = –1.66–5.40%) T (8) = 0.50, *p* = .63 (Figure 5). Her hippocampal atrophy rate, however, was similar to those of the three *MAPT* cases of comparable disease duration. Therefore, CW’s hippocampal and whole-brain atrophy rates were not atypical compared to other *MAPT* mutation cases in our FTD cohort.

## DISCUSSION

Here we report a novel *MAPT* mutation case in exon 12 with a predominantly amnestic presentation masquerading as AD initially. The mutation causes a glutamine to arginine substitution at codon 351 (Q351R) in the fourth microtubule repeat domain with a PolyPhen-2 score of 0.891, i.e. possibly damaging (Adzhubei et al., 2010). The substitution is likely to lead to reduced capacity of tau protein to bind to microtubules and, or increased fibrillogenicity (Hong et al., 1998; Spillantini, Van Swieten, & Goedert, 2000). Given the long duration of the case and the suggestion that disease severity of *MAPT* mutations are in part related to the relative reduction of the ability of the tau protein to interact with microtubules (Spillantini et al., 2000; Van Swieten et al., 1999), *in vivo* studies assessing tau functions in this mutation would be particularly instructive.

The key clinical features of *MAPT* mutations are behavioral changes, semantic impairment, episodic memory decline, and parkinsonism (Rohrer & Warren, 2011). Although behavioral changes are the prototypical presenting symptoms of *MAPT* cases (Rohrer et al., 2011), any of the other nonbehavioral features may lead (Doran et al., 2007; Ishizuka, Nakamura, Ichiba, & Sano, 2011; Larner, 2008; Rohrer et al., 2011). When episodic memory impairment is prominent, it is not uncommon for AD to be diagnosed. This has been reported for mutations involving both the coding regions (Mirra et al., 1999) (Tolboom et al., 2010) and the intronic regions (Doran et al., 2007; Larner, 2008). As highlighted in a recent review (Hornberger & Piguet, 2012), the magnitude of anterograde memory deficits in FTD can be very similar to that in AD. Our case illustrates this point and the importance of screening for *MAPT* mutations in families with an autosomal dominant history of an amnestic syndrome where mutations for familial AD have proven negative (Larner, 2008).

It is well established that the same *MAPT* mutation can give rise to different clinical phenotypes. For example, the P301L mutation has been reported to present with amnesia, behavioral change, semantic loss, or motor syndromes (Donker Kaat et al., 2009; Ishizuka et al., 2011; Mirra et al., 1999; Van Swieten et al., 1999). It has led to the suggestion that molecular pathologies do not necessarily specify a precise initial neuroanatomical locus of damage (Rohrer et al., 2010); other factors such as developmental vulnerability or environmental factors may possibly influence this (Mesulam, 2009). It remains to be seen whether other clinical phenotypes of the Q351R mutation would emerge in due course.

A significant aspect of the current case was the initial diagnostic difficulty. During the early years, there was a suggestion of a significant functional component to CW’s presentation. It was thought that her very poor performance on tests of memory and confrontation naming were inconsistent with her fluent, articulate speech and reasonable account of day-to-day events. However, as naming deficits are very rarely a cognitive correlate of depression (Emery & Breslau, 1989), in retrospect, her reduced confrontation naming—present even at the initial assessment—should have pointed to a neurogenic cognitive impairment suggesting early subtle semantic impairment which perhaps should have raised the suspicion of a non-AD diagnosis at the time. As mentioned earlier, semantic impairment is a frequent finding in cases of *MAPT* mutations (Rohrer & Warren, 2011; Seelaar et al., 2008) and can be present early or even in the presymptomatic phase of the illness (Garrard & Carroll, 2005; Ishizuka et al., 2011). Early profound anomia was also present in a family with an intron 10 + 16 mutation, many of whom presented with amnestic symptoms (Doran et al., 2007). Other evidence of semantic degradation in CW, including reduced semantic fluency and poor synonym-matching scores, emerged over time (Table 1). Interestingly, apart from the observation of poverty of speech content, no major concern with her language function has been raised at any time.

Similarly CW’s poor performance on the retention trials of the Gollin figure test was seen as supportive evidence of a significant functional component as it was assumed that even amnestic patients would usually show some implicit learning (Warrington & Weiskrantz, 1968). The most likely explanation is that her profoundly impaired episodic memory system, together with weakened support from the visual semantic system resulted in an inability to support such implicit learning. There is little doubt that other apparent discrepancies between her scores on memory tests and her clinical presentations were over emphasized. This illustrates the difficulty in making such judgements based on relatively brief clinical contact.

Both MRI and CSF played a role in making the eventual diagnosis. Progressive medial temporal lobe atrophy along an anterior–posterior gradient provided a useful diagnostic pointer (Chan et al., 2001). Arguably, MR techniques such as fluid registration allow more focal measurements and regional visualization so could potentially detect these changes before routine radiological review. At the time of initial investigation and CSF examination, tau and Aβ_42_ protein assays were not available. When reinvestigation—10 years later—was prompted by the atypical clinical course and the MRI findings, these assays had been established and lent support to a non-AD pathology.

For the last few years, CW has given a very circumscribed and highly stereotyped account of her autobiographical history. Her extremely limited repertoire of autobiographical accounts is unlike that typically seen in patients with either AD or semantic dementia (SD) (Graham & Hodges, 1997; Leyhe, Müller, Milian, Eschweiler, & Saur, 2009) and is likely to be the result of an interaction of her impaired episodic and semantic memory systems with additional contribution from impaired strategic retrieval mechanisms (Matuszewski et al., 2006). Autobiographical memory (AbM) has both an episodic component and a personal semantic memory component (Tulving, 1993). In AD, personal semantics scores correlate with bilateral anterior and posterior lateral temporal lobe volume and episodic AbM scores correlate with combined atrophy in bilateral medial temporal lobes and anterior lateral temporal neocortex (Gilboa et al., 2005). The impairment CW shows in both components of AbM is consistent with the neuroimaging findings of severe temporal lobe atrophy involving the anterior and medial aspects but also, to a lesser extent, the lateral temporal cortex. In terms of a temporal gradient, patients with early AD typically remember remote personal facts and incidents better than recent ones (maintenance of the reminiscence bump and absence of the “recency” effect) (Morris & Mograbi, 2013). The opposite pattern is seen in early SD (Graham & Hodges, 1997; Hou, Miller, & Kramer, 2005; Irish et al., 2011) although as disease progresses, memory for all time periods is degraded (Matuszewski et al., 2009). Interestingly, CW appeared to have slightly better recall of the AbM for early adulthood compared with childhood and recent life, namely a relatively preserved reminiscence bump, but with no other temporal gradient in either direction. The autobiographical memory interview (Kopelman et al., 1989) used in our study has a free recall and a general probing condition. A more detailed procedure such as one that employs specific probes could potentially yield more informative findings (Irish et al., 2011). Systematic study of the organization of AbM in patients with genetic FTD is needed to shed further light on this relatively little investigated area.

CW’s serial volumetric MR images over a 12-year period and fluid registration of her MRI brain scans demonstrate a relatively symmetrical pattern of atrophy affecting the frontal, temporal and, to a lesser extent, parietal lobes with particular emphasis on the anterior, medial, and inferior aspects of the temporal lobes (Figures 1–3). This concurs with previous MRI findings of bilateral anteromedial temporal lobe involvement in *MAPT* mutation (Rohrer et al., 2010 2011; Whitwell et al., 2009 2012). In contrast with Whitwell et al.’s findings that mutations which affect the structure of the tau protein preferentially involve the lateral temporal lobes with relative sparing of the medial temporal lobe (Whitwell et al., 2009), for this novel mutation with putative defect in tau structure, the medial temporal lobes are more atrophied.

The remarkably slow progression of this case is instructive for the discussion on FTD phenocopy syndrome (Kipps, Hodges, & Hornberger, 2010; Piguet, Hornberger, Mioshi, & Hodges, 2011) and suggests a need for caution in the diagnosis in such cases. On the basis of the slow pace of progression, similar cases, particularly those led by behavioral problems might have been diagnosed as phenocopies. As noted earlier, compared with other *MAPT* cases, during the early years, CW’s whole-brain atrophy rate was toward the lower end of the range whilst her hippocampal atrophy rate was toward the higher end. This could suggest that the overall rate of clinical progression may be better correlated with the extent to which pathology spreads outside the initial focus. A caveat of making such comparisons with other *MAPT* mutation cases is that we were limited by the number of cases in the local FTD cohort with comparable disease duration.

In conclusion, despite the initial atypical presentation of the case, subsequent development of semantic deficits and behavioral changes as well as the MRI atrophy patterns are consistent with existing literature on *MAPT* mutations. This supports the hypothesis that in *MAPT* mutations, although the initial target of the disease may be stochastic, the subsequent propagation is likely to conform to a specific, intrinsic brain network according to the underlying molecular pathology (Warren, Rohrer, & Hardy, 2012).

This case also illustrates a number of clinical and neuropsychological issues: the significance of anomia in the context of atypical amnesia in pointing toward a non-AD diagnosis; the value of searching for a *MAPT* mutation in cases of early onset dementia characterized by amnesia and relevant family history with negative familial AD mutations; the complexity in differentiating organic and functional amnesia and the unique effect on autobiographical memory as a result of an interaction between damaged episodic and semantic memory systems.
